# Balneotherapy for Fibromyalgia Syndrome: A Systematic Review and Meta-Analysis

**DOI:** 10.3390/jcm10071493

**Published:** 2021-04-03

**Authors:** Chun-Feng Cao, Kun-Long Ma, Qian-Lu Li, Fu-Jun Luan, Qun-Bo Wang, Ming-Hua Zhang, Omar Viswanath, Dariusz Myrcik, Giustino Varrassi, Hai-Qiang Wang

**Affiliations:** 1 Department of Orthopedics, The Yongchuan Hospital of Chongqing Medical University, 439# Xuanhua Road, Yongchuan, Chongqing 402160, China; 711204@hospital.cqmu.edu.cn (C.-F.C.); 701079@hospital.cqmu.edu.cn (K.-L.M.); 701148@hospital.cqmu.edu.cn (F.-J.L.); 700009@hospital.cqmu.edu.cn (Q.-B.W.); 700280@hospital.cqmu.edu.cn (M.-H.Z.); 2 Department of Neurology, The Yongchuan Hospital of Chongqing Medical University, 439# Xuanhua Road, Yongchuan, Chongqing 402160, China; 710197@hospital.cqmu.edu.cn; 3 Department of Anesthesiology, Creighton University School of Medicine, Omaha, NE 68114, USA; viswanoy@gmail.com; 4 Department of Anesthesiology, University of Arizona College of Medicine-Phoenix, Phoenix, AZ 85003, USA; 5 Department of Anesthesiology, Louisiana State University Shreveport, Shreveport, LA 71106, USA; 6 Department of Pain Management, Valley Pain Consultants-Envision Physician Services, Phoenix, AZ 85003, USA; 7 Department of Internal Medicine, Medical University of Silesia, Katowice, 42-600 Bytom, Poland; dariuszmyrcik@me.com; 8 Department of Research, Polo Procacci Foundation, via Tacito 7, 00193 Roma, Italy; 9 Institute of Integrative Medicine, Shaanxi University of Chinese Medicine, Xixian Avenue, Xixian District, Xi’an 712046, China

**Keywords:** balneotherapy, fibromyalgia, meta-analysis, systematic review, musculoskeletal disease

## Abstract

(1) Background: The efficiency of balneotherapy (BT) for fibromyalgia syndrome (FMS) remains elusive. (2) Methods: Cochrane Library, EMBASE, MEDLINE, PubMed, Clinicaltrials.gov, and PsycINFO were searched from inception to 31 May 2020. Randomized controlled trials (RCTs) with at least one indicator were included, i.e., pain, Fibromyalgia Impact Questionnaire (FIQ), Tender Points Count (TPC), and Beck’s Depression Index (BDI). The outcome was reported as a standardized mean difference (SMD), 95% confidence intervals (CIs), and *I*^2^ for heterogeneity at three observational time points. GRADE was used to evaluate the strength of evidence. (3) Results: Amongst 884 citations, 11 RCTs were included (*n* = 672). Various BT regimens were reported (water types, duration, temperature, and ingredients). BT can benefit FMS with statistically significant improvement at different time points (pain of two weeks, three and six months: SMD = −0.92, −0.45, −0.70; 95% CI (−1.31 to −0.53, −0.73 to −0.16, −1.34 to −0.05); *I*^2^ = 54%, 51%, 87%; GRADE: very low, moderate, low; FIQ: SMD = −1.04, −0.64, −0.94; 95% CI (−1.51 to −0.57, −0.95 to −0.33, −1.55 to −0.34); *I*^2^ = 76%, 62%, 85%; GRADE: low, low, very low; TPC at two weeks and three months: SMD = −0.94, −0.47; 95% CI (−1.69 to −0.18, −0.71 to −0.22); *I*^2^ = 81%, 0; GRADE: very low, moderate; BDI at six months: SMD = −0.45; 95% CI (−0.73 to −0.17); *I*^2^ = 0; GRADE: moderate). There was no statistically significant effect for the TPC and BDI at the remaining time points (TPC at six months: SMD = −0.89; 95% CI (−1.85 to 0.07); *I*^2^ = 91%; GRADE: very low; BDI at two weeks and three months: SMD = −0.35, −0.23; 95% CI (−0.73 to 0.04, −0.64 to 0.17); *I*^2^ = 24%, 60%; GRADE: moderate, low). (4) Conclusions: Very low to moderate evidence indicates that BT can benefit FMS in pain and quality-of-life improvement, whereas tenderness and depression improvement varies at time phases. Established BT regimens with a large sample size and longer observation are needed.

## 1. Introduction

Fibromyalgia syndrome (FMS) is a musculoskeletal disorder characterized by widespread skeletal muscle pain. It is also considered a chronic pain syndrome mainly due to the dysfunction of the central nervous system [1]. Patients can present with various accompanied symptoms, including fatigue, sleep disturbances, headache, morning stiffness, anxiety, and depression [2,3]. The prevalence of FMS is estimated at 1–2% in the general population. The disorder mainly affects women, with an incidence rate six times higher than that of men [4]. Its treatment is usually complex [5] and unsatisfactory, thus being identified as a public health burden due to significant health expenses for the treatment of the musculoskeletal disease, including FMS [6]. Consequently, FMS patients are responsible for a high rate of medical consultations and a high consumption of drugs [7].

Balneotherapy (BT) is a non-invasive alternative treatment to relieve musculoskeletal or neuropathic pain and stiffness, improving the quality of life amongst the elderly with musculoskeletal pain [8]. Despite BT being defined as bathing in natural mineral waters, spas, or cures as a Medical subject Headings (MeSH) term (MeSH Unique ID: D001452), the practice mode varies greatly in the literature, including the temperature of the waters, therapy duration/sessions, mineral contents, and concentrations [9,10,11]. Previous randomized clinical trials (RCTs), meta-analyses, and systematic reviews have shown its effectiveness in alleviating symptoms of patients with musculoskeletal disorders [12,13]. However, studies on BT for FMS are disputable with a potential source of bias, such as a lack of double-blinded studies [14,15]. So far, there has been a paucity of randomized evidence investigating the efficiency of BT for FMS. A previous study has evaluated the issue, with limited outcome indicators [9]. Moreover, new studies on BT for FMS have not been successfully analyzed by systematic reviews until now. Therefore, we conducted an updated systematic review and meta-analysis to determine whether the existing data show the efficacy of BT in the treatment of FMS.

## 2. Methods

Given that the study was a systematic review, ethics committee approval was waived.

### 2.1. Protocol and Registration

This systematic review and meta-analysis was performed according to the Preferred Reporting Items for Systematic Review and Meta-Analysis Protocols 2015 (PRISMA-P) guidelines [16]. It has been registered in the International Prospective Register of Systematic Reviews (PROSPERO), with the registration number CRD42019142187.

### 2.2. Search Strategy

We searched the Cochrane Library, EMBASE, MEDLINE, PubMed, Clinicaltrials.gov, and PsycINFO thoroughly from their inception to 31 May 2020 to quantitatively compare the pooled effect of BT for FMS. The searching strategies were worked out by two reviewers (C.F.C. and K.L.M.) who were experienced to identify relevant studies. We adopted the search strategies “(FMS OR fibromyalgia OR musculoskeletal disease) AND (BT OR spa therapy OR balneotherapy OR balneology OR thermal water)” without language restrictions (Table 1). The search filter was limited to randomized clinical trials (RCTs). In addition, the reference lists of relevant articles were reviewed for further papers to be included.

### 2.3. Inclusion and Exclusion Criteria

Studies were required to meet the following inclusion criteria: (1) RCTs with FMS diagnosis based on the American College of Rheumatology (ACR) criteria [17,18]; (2) type of intervention as comparing BT with no treatment or other treatment options; (3) outcome measures reporting at least one FMS symptom-related item, including pain, Tender Points Count (TPC), Beck’s Depression Index (BDI), and Fibromyalgia Impact Questionnaire (FIQ); and (4) publication of the study in full paper form.

We excluded retrospective studies, cohort studies, and clinical controlled studies. Two authors (C.-F.C. and K.-L.M.) screened the studies independently. An initial screening of titles was performed, with duplicates excluded. Subsequently, full texts and abstracts were reviewed and irrelevant papers were removed.

### 2.4. Data Extraction and Management

Data were extracted from all the studies meeting the inclusion criteria, and following an instruction manual. For all articles, two authors (Q.-L.L. and F.-J.L.) reviewed data independently. Potential disagreements were solved by consensus. When consensus could not be reached, a third author (M.-H.Z.) was required to extract data and discuss for consensus. When data were reported as a median (low–high), the mean and variance were calculated using the appropriate formula [19].

### 2.5. Assessment of Risk of Bias (ROB) in Included Studies

Two authors (C.-F.C. and K.-L.M.) assessed the ROBs of each study independently according to the Cochrane Collaboration’s ROB 2 tool [20]. ROB 2 includes optional judgments of the direction of bias for each domain and overall. We applied the form of ROB assessment, including five key indicators: randomization process, intended interventions, missing outcome data, the measurement of the outcome, and the selection of reported results. All disagreements were resolved by consensus and eventually the consultation of a third author (Q.-B.W.).

### 2.6. Assessment of Treatment Effect

Meta-analysis was performed using Review Manager 5.3 software. The standardized mean difference (SMD) was calculated using a random-effects model, when data were continuous, and 95% confidence intervals (CIs) were determined for all effect sizes. The strength of evidence was assessed using the online version of GRADEpro GDT software, identified as high, moderate, low, and very low [21].

### 2.7. Assessment of Heterogeneity

Heterogeneity between comparable trials was analyzed using standard Cochran’s Q tests and the *I*^2^ statistic before meta-analysis. *I*^2^ values were taken according to Deeks [22]. A *p*-value of < 0.05 was considered statistically significant.

### 2.8. Sensitivity Analysis

Sensitivity analysis was performed for high-risk and low-risk bias studies, studies with serious deficiencies in one or more key areas, and the sample size for each treatment group.

## 3. Results

### 3.1. Hallmarks of Included Studies

**Figure 1:**
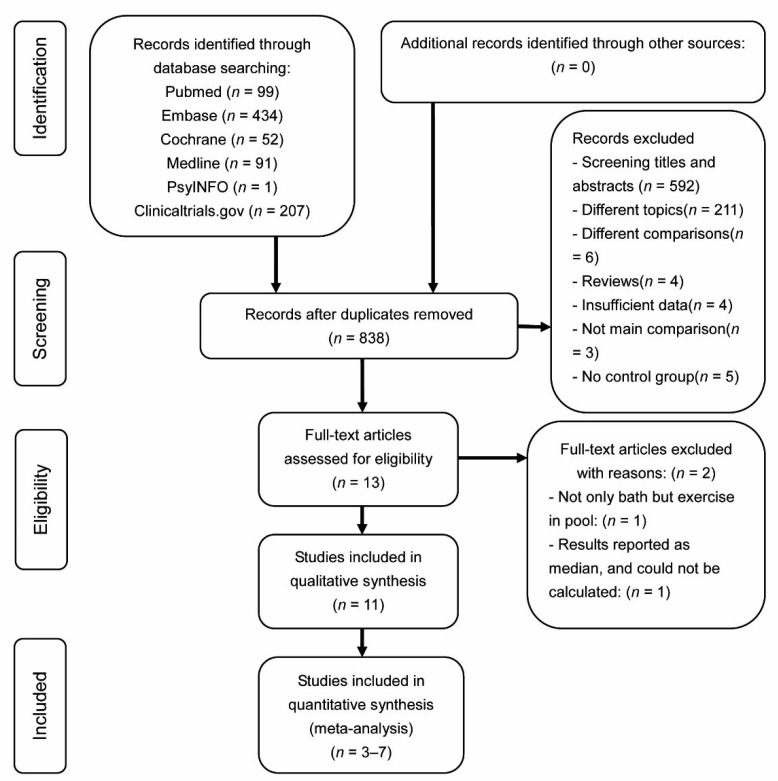
Flowchart of literature-screening process.

The literature-retrieving strategy and pertaining results are shown in Figure 1. A total of 884 relevant studies were preliminarily reviewed (PubMed search, 99 citations; Cochrane search, 52 citations; EMBASE search, 434 citations; Medline search, 91 citations; PsycINFO search, 1 citation; and Clinicaltrail.gov search, 207 citations). In total, 13 RCT studies eventually satisfied the eligibility criteria, and 11 were included for this meta-analysis. After the initial screening, one study was excluded, given that the means and standard deviation of post-test data were not reported and could not be calculated [23]. Another study was excluded because the exercises in the pool were used as therapy, and not only the bath [24].

### 3.2. Location Hallmarks

Included RCTs were mainly derived from Europe and Asia. In detail, six studies were from Turkey [8,25,26,27,28,29], three from Italy [30,31,32], one from Spain [33], and one from the Netherlands [34].

### 3.3. Participant Hallmarks

In total, 672 participants were included, with 330 patients undergoing BT intervention and 342 being controls. The average age was 46.1 years in the BT group (range, 42.0 to 56.2 years) and 45.7 years in the control group (range, 41.5 to 55.9 years). The gender was reported in nine studies. Females were highly prevalent (287 females and 15 males in the BT group, 306 females and 14 males in the control group). The average observational time phase was 19.7 weeks (range, 3 to 48 weeks). The average disease duration was 5.13 years according to data in nine studies (range, 1.3 to 12.9 years). A detailed list of study hallmarks is shown in Table 2.

### 3.4. ROBs of Included Studies

**Figure 2:**
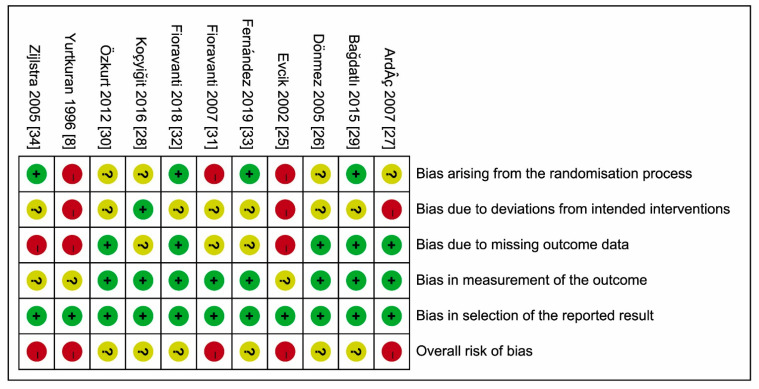
Risks of bias (ROBs) (2.0) within the included studies. Green circle and ‘+’, low risk; red circle and ‘−’, high risk; yellow circle and ‘?’, unclear risk.

None of the 11 studies had low ROBs (Figure 2); 6 had some concerns (unclear intended interventions [28,32], unclear intended interventions [26,29] and randomization process [30], unclear intended interventions and missing outcome data [33]), while the remaining 5 studies had high ROBs [8,25,27,31,34].

### 3.5. Publication Bias

**Figure 3:**
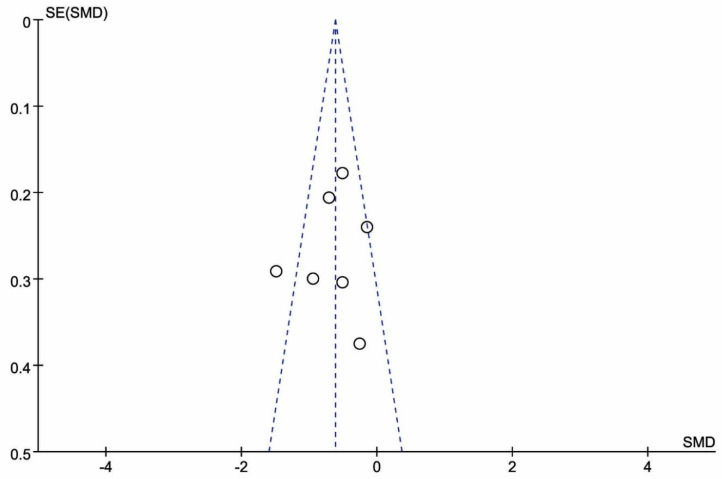
Funnel plot of the Fibromyalgia Impact Questionnaire (FIQ) at three months following treatment with BT. The following visual analysis of funnel plots revealed symmetric images, with robust-to-potential publication bias.

Given that the visual analysis of funnel plots (Figure 3) revealed symmetric images, the results of the meta-analysis were considered robust-to-potential publication bias (Egger’s test, *p* = 0.66).

### 3.6. Effects of BT Interventions

#### 3.6.1. BT Intervention Sessions and Data Analysis Time Points

Amongst 11 included studies, most BT intervention sessions were 2 weeks (8 studies), whereas 2 studies were 3 weeks, and 1 study was 2.5 weeks. A detailed list of BTs (including water temperature, therapy time, therapy session, and components) is shown in Table 3. There were nine types of BT water and seven types as control treatment. The average BT duration was 20.5 min (range, 15–30 min; three types). The average BT water temperature was 36.5 (range, 34.8–38 °C; five types). There were six types of ingredients for BT in six studies.

For pooled analysis, we included the eight studies with outcome measures using two-week sessions as treatment completing time point, corresponding studies with measures at three months as follow-up time point, and studies with measures at six months as final effect time point.

#### 3.6.2. Primary Outcome Measures

##### Pain

**Figure 4:**
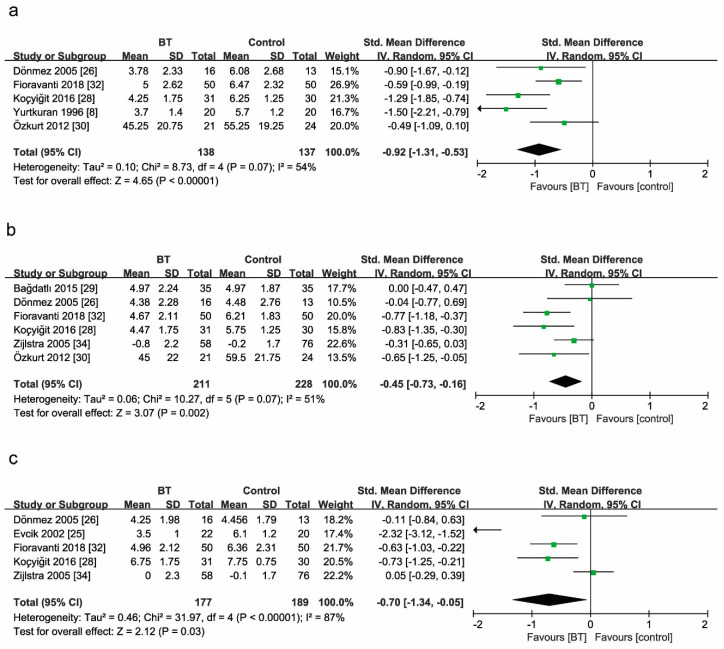
Forest plot for the comparison of pain (**a**) at 2 weeks, showing the effect favoring balneotherapy; (**b**) at 3 months, showing the effect favoring balneotherapy; and (**c**) at 6 months, showing the effect favoring balneotherapy. Green square represent the std. mean difference, bars represent the 95% confidence interval, and black diamond represent the pooled analysis for each pain score.

Five studies reported the comparison of pain between BT and control groups at two weeks as the end of BT sessions (Figure 4a). The pain scores in the BT group were 92% lower than those in the control group (SMD = −0.92, 95% CI (−1.31 to −0.53), *p* < 0.00001). Test statistics revealed considerable heterogeneity among these studies (*I*^2^ = 54%). The comparison was rated as very low quality evidence by GRADE (Table 4).

Six studies reported the comparison of pain between BT and control groups at three months (Figure 4b). BT reduced the pain score by 45% (SMD = −0.45, 95% CI (−0.73 to −0.16), *p* = 0.0002; GRADE: moderate). Considerable heterogeneity existed among these studies (*I*^2^ = 51%).

Five studies reported the comparison of pain between BT and control groups at six months (Figure 4c). BT reduced the pain score by 70% (SMD = −0.70, 95% CI (−1.34 to −0.05), *p* = 0.03; GRADE: low). Considerable heterogeneity existed among these studies (*I*^2^ = 87%).

##### Fibromyalgia Impact Questionnaire

**Figure 5:**
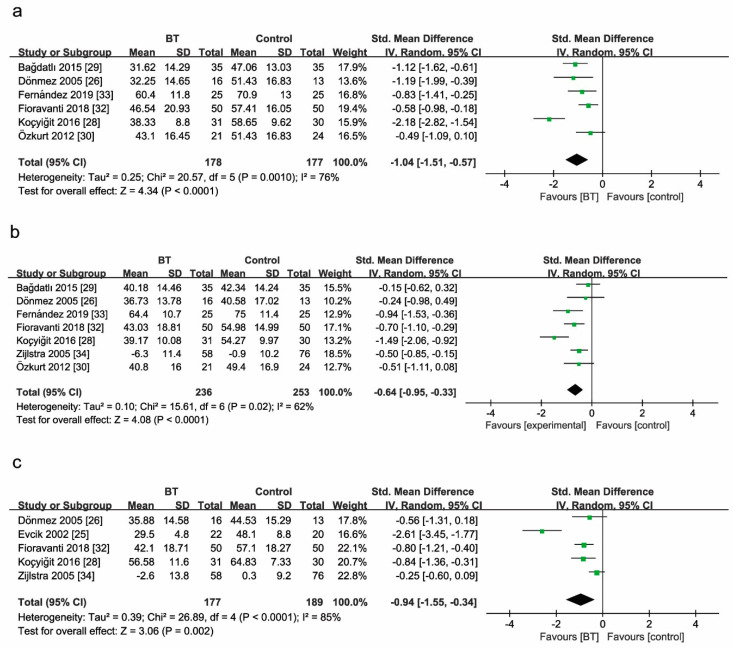
Forest plot (**a**) for the comparison of the FIQ score at 2 weeks, showing the effect favoring balneotherapy; (**b**) at 3 months, showing the effect favoring balneotherapy; and (**c**) at 6 months, showing the effect favoring balneotherapy. In the figure, the mean indicates mean FIQ values. Green square represent the std. mean difference, bars represent the 95% confidence interval, and black diamond represent the pooled analysis for each FIQ score.

Of the 11 RCTs, 6 used the FIQ at two weeks (Figure 5a). The mean values in Figure 5 indicated FIQ scores at the same observational time after treatment as listed for each group (BT group and control group). BT improved the FIQ score by 104% when compared with controls (SMD = −1.04, 95% CI (−1.51 to −0.57), *p* < 0.00001; GRADE: low). Considerable heterogeneity was identified among these studies (*I*^2^ = 76%).

Seven studies reported the comparison of the FIQ score between BT and control groups at three months (Figure 5b). BT improved the FIQ score by 64% (SMD = −0.64, 95% CI (−0.95 to −0.33), *p* < 0.00001; GRADE: low). Considerable heterogeneity existed among these studies (*I*^2^ = 62%).

Five studies reported the comparison of the FIQ score between BT and control groups at six months (Figure 5c). Analogously, pooled results of subgroup analysis indicated that BT improved the FIQ score by 94% when compared with controls (SMD = −0.94, 95% CI (−1.55 to −0.34), *p* = 0.002; GRADE: very low). Considerable heterogeneity presented among these studies (*I*^2^ = 85%).

##### Tender Points Count

**Figure 6:**
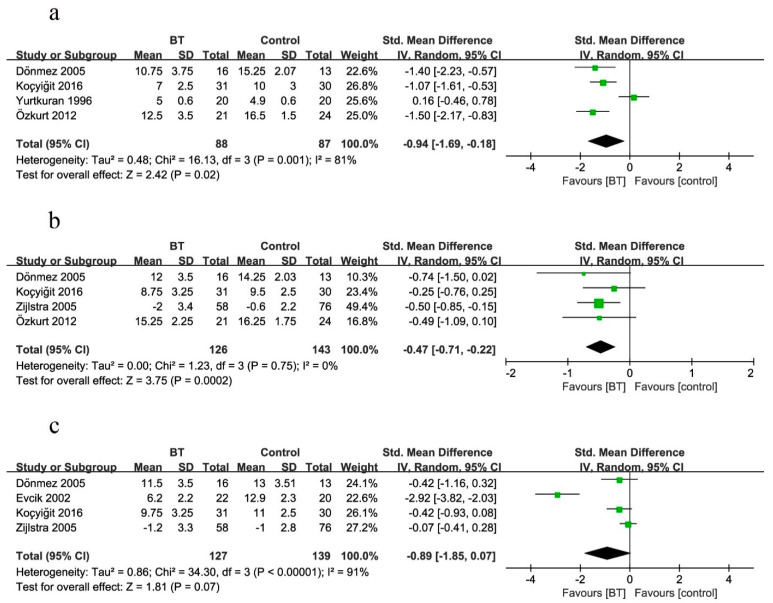
Forest plot for the comparison of the TPC (**a**) at 2 weeks, showing the effect favoring balneotherapy; (**b**) at 3 months, showing the effect favoring balneotherapy; and (**c**) at 6 months, showing the effect not favoring balneotherapy. In the figure, the mean indicates mean TPC values. Green square represent the std. mean difference, bars represent the 95% confidence interval, and black diamond represent the pooled analysis for each TPC.

Four studies measured the TPC as the outcome at two weeks (Figure 6a). The mean values in Figure 6 indicated the TPC at the same observational time after treatment as listed for each group (BT group and control group). BT improved the clinical efficacy of the TPC by 94% when compared with controls (SMD = −0.94, 95% CI (−1.69 to −0.18), *p* = 0.02; GRADE: very low). Considerable heterogeneity existed among these studies (*I*^2^ = 81%).

Four studies measured the TPC as the outcome at three months (Figure 6b). BT improved the clinical efficacy of the TPC by 47% when compared with controls (SMD = −0.47, 95% CI (−0.71 to −0.22), *p* = 0.0002; GRADE: moderate). No heterogeneity existed (*p* = 0.75, *I*^2^ = 0%).

Four studies reported the comparison of the TPC between BT and control groups at six months (Figure 6c). There was no statistical difference effect on the TPC (SMD = −0.89, 95% CI (−1.85 to 0.07), *p* = 0.07; GRADE: very low). Considerable heterogeneity was noted among these studies (*I*^2^ = 91%).

##### Beck’s Depression Index

**Figure 7:**
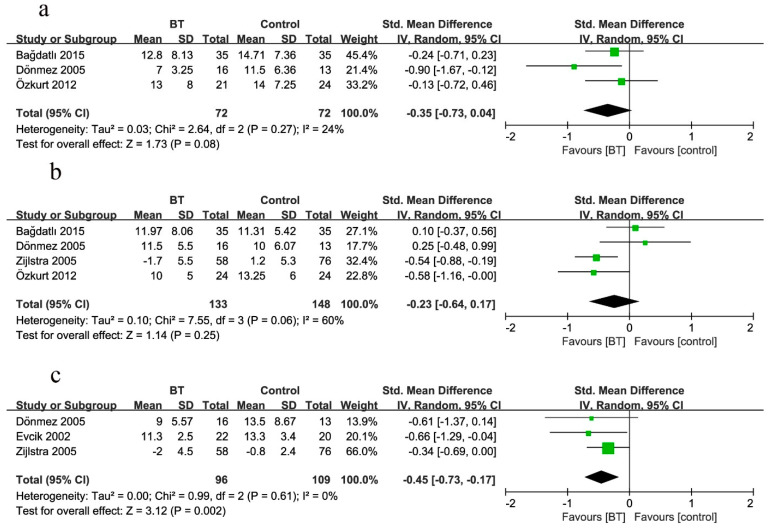
Forest plot for the comparison of the BDI (**a**) at 2 weeks, showing the effect not favoring balneotherapy; (**b**) at 3 months, showing the effect not favoring balneotherapy; and (**c**) at 6 months, showing the effect favoring balneotherapy. Green square represent the std. mean difference, bars represent the 95% confidence interval, and black diamond represent the pooled analysis for each BDI.

Three studies measured the BDI as the outcome at two weeks (Figure 7a). There was no statistical difference effect on the BDI (SMD = −0.35, 95% CI (−0.73 to 0.04), *p* = 0.06; GRADE: moderate), with no heterogeneity (*p* = 0.27, *I*^2^ = 24%).

Four studies measured the BDI as the outcome at three months (Figure 7b). Analogously, there was no statistical difference effect on the BDI (SMD = −0.23, 95% CI (−0.64 to 0.17), *p* = 0.25; GRADE: low). Considerable heterogeneity was noted among these studies (*I*^2^ = 60%).

Three studies reported the comparison of the BDI between BT and control groups at six months (Figure 7c). BT improved the BDI by 45% when compared with controls at the end of treatment (SMD = −0.45, 95% CI (−0.73 to −0.17), *p* = 0.002; GRADE: moderate), with no heterogeneity (*p* = 0.61, *I*^2^ = 0%).

### 3.7. Sensitivity Analyses

**Figure 8:**
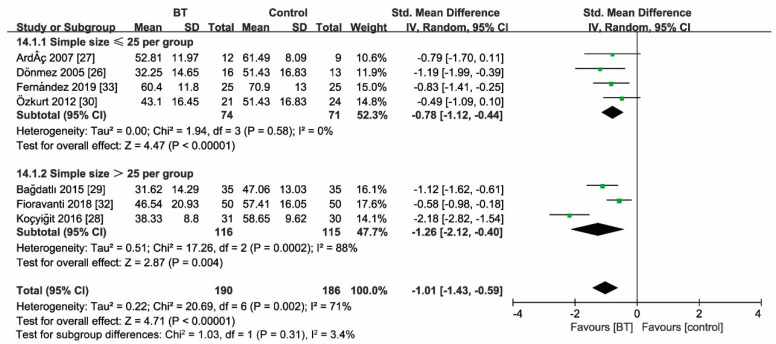
Sensitivity analysis for the sample size (FIQ at 2 weeks), indicating unfaltering FIQ efficacy at 2 weeks after treatment. Green square represent the std. mean difference, bars represent the 95% confidence interval, and black diamond represent the pooled analysis for FIQ score at 2 weeks in different simple size group.

**Figure 9:**
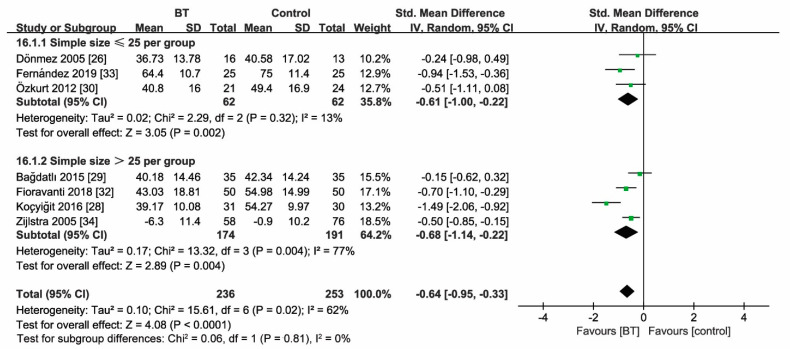
Sensitivity analysis for the sample size (FIQ at 3 months), showing unfaltering FIQ efficacy at 3 months after treatment. Green square represent the std. mean difference, bars represent the 95% confidence interval, and black diamond represent the pooled analysis for FIQ score at 3 months in different simple size group.

Sensitivity analyses according to sample size (*n* ≤ 25, >25) revealed unaltered outcome measures as the FIQ score at two weeks and three months after treatment (Figure 8 and Figure 9). Moreover, statistical heterogeneity of analysis for the effect size of the FIQ score in two weeks (*I*^2^ = 71%) and three months (*I*^2^ = 62%) substantially decreased (two weeks: *I*^2^ = 0%; three months: *I*^2^ = 12%) by removing the study of Koçyiğit et al. The magnitude of the effect size decreased (two weeks: SMD = 0.78; 95% CI (−1.01, −0.55), *p* < 0.00001; three months: SMD = 0.52; 95% CI (−0.73, −0.31), *p* < 0.00001).

## 4. Discussion

The systematic review and meta-analysis first addressed the effect of BT on FMS at triple time points with a clear level of evidence reflected by GRADE. Amongst 672 participants, very low to moderate evidence indicates that BT can benefit FMS in pain and quality-of-life improvement, whereas tenderness and depression improvement is uncertain at different time phases.

Although guidelines or recommendations are available for the management of FMS, they are mostly based on expert consensus with some limitations [35]. European League Against Rheumatism (EULAR) recommendations for the management of FMS [5] were updated in 2017 based on systematic reviews. The importance of the multidisciplinary approach was highlighted, with special emphasis on non-pharmacological treatments for FMS. It should be stressed that BT was largely neglected in the EULAR recommendations for FMS, which used the term hydrotherapy/spa therapy. According to the nomenclature as a MeSH term, BT is not equal to or the same as hydrotherapy or spa therapy. Neumann’s study [12] is the only available meta-analysis on BT in the EULAR recommendations, covering the literature up to April 2013. Six RCTs with 311 participants were analyzed for their meta-analysis, including 149 patients in the BT group and 162 individuals in the control group. Without a GRADE basis, they concluded moderate evidence of a medium-to-large effect on pain and the TPC for BT, a medium effect on the FIQ score, and no significant effect on the BDI. Importantly, outcome measures were ambiguous in that meta-analysis. All outcomes were analyzed in the final treatment and at follow-up, but the time frames were not available. In fact, the treatment sessions of BT were 2.5 weeks in Zijlstra [34] and 3 weeks in Evcik [25] and Ardıç [27]. Zijlstra et al. [34] provided Visual Analogue Scale (VAS) pain data at 3, 6, and 12 months after treatment. Only the data of six months of follow-up were included in their study [12]. Moreover, the TPC and FIQ data at three months were included in the previous study [12]. Thereafter, there have been no latest systematic reviews regarding the topic during the past six years.

This meta-analysis provided recent evidence for the potential treatment of FMS, including 11 selected studies with a total of 672 participants. The updated four studies greatly expanded the number of participants, significantly improving the reliability of the meta-analysis results. Moreover, we extracted all informative data from original RCTs, which may significantly improve reliability. The time points for the extracted data set were at two weeks after the start of treatment, three months, and six months as the final treatment.

Our findings are consistent with previous meta-analyses or reviews [12,13,15]. Notably, we found very low and moderate evidence that BT can benefit FMS with the TPC at two weeks and three months, but there is no significant effect at six months. This point is contrary to a previous meta-analysis [12] or the others where authors neglected the data on the TPC at follow-up (three months) [30].

Three included studies adopted the 100 mm Visual Analog Scale [26,30,31]. A significant improvement of pain connected with FMS was registered. The degrees of pain relief vary among the included studies, with different baselines. Functional capacity in daily-living activities were evaluated by the FIQ [36]. The results of this meta-analysis showed that BT can significantly improve functional outcomes. Interestingly, the efficacy of BT decreased with long-lasting treatment, although it was still effective in the last follow-up (24 weeks). However, this interesting finding was not observed in Altan’s study [37]. Probably, this was due to the absence of subsequent treatment after BT able to maintain persistent efficacy. Similarly, the current study found that the TPC significantly reduced in the bathing treatment group in comparison with the control group. There was no significant effect on the TPC at six months. This finding is also consistent with previous studies [38,39], which demonstrated that BT has a significant effect on the improvement of the TPC. It is possible that the function of BT in the elimination of inflammatory factors reached an extreme in a certain period of treatment time [40]. Consequently, FMS patients can still choose BT as an early treatment option. Beyond that time, the pain relief from BT cannot continue. Therefore, the FMS patient’s adherence to longer treatment is needed to continue the effect [41], and maybe intermittent safe administration of non-steroidal anti-inflammatory drugs would help [42]. Improvement in mental health was less pronounced and quick in physical health, suggesting that BT exerts predominantly physical effects. As pain and the quality of life improve, depression slowly improves.

The underlying mechanisms of BT effects can be due to heat, mineral content, and other physiologic and endocrine effects [43,44]. Thermal stress stimulation exerts analgesic effects on nerve endings by increasing the pain threshold. It alleviates muscle spasms and activates the pain-relieving inhibition system through the gamma fibers of muscle spindles. According to gate theory, pain relief may be caused by water temperature and pressure on the skin [45,46]. Physiologically, heat application leads to increased blood circulation, and heat application to inflamed tissue induces oxygen free radical removal and enhances the repair of the inflammatory tissue [47,48,49]. In different musculoskeletal diseases, the effect of BT on pain and function is significantly better and longer than that of a tap water bath at the same temperature [50]. The minerals that are dissolved in the water play an important part in the mechanism of action of BT. However, there have been no established criteria for these important parameters regarding BT for FMS.

High heterogeneity was noted between included studies due to several factors. First, observational phases varied, as listed in Table 2. The last follow-up time after intervention is an important factor in determining the sustained effectiveness of BT. Notably, exposure times and temperature for BT varied. Second, the composition of the mineral water and the place of spa therapy were different. Third, the definition of quality of life was not consistent due to different economic levels and regional cultures. Finally, due to the special hallmarks of the intervention, almost all included randomized controlled studies were not perfect in terms of the blind methodology. These factors may be contributive to the inter-group differences, affecting the entire heterogeneity of included studies.

This meta-analysis had several limitations. The sample sizes in the included studies were small, and this was a confusing bias. Moreover, there were no unified evidence-based diagnostic regimens for included studies, including mechanisms, common characteristics, and comorbidities, capable of improving the recognition of FMS in clinical practice. Therefore, we tried to choose randomized control trials based on FMS guidelines. Furthermore, this meta-analysis including 11 RCTs could not identify the underlying factors leading to heterogeneity. Additional studies to perform subgroup analyses are needed in the future.

## 5. Conclusions

This meta-analysis highlights there is, in fact, a role that BT can and should play in the complex treatment of FMS. In patients dealing with FMS, any opportunity to improve daily functionality and reduce chronic pain is a welcome addition to the treatment regimen. We hope this study will propagate further discussion with the consideration of the implementation of BT in the treatment for patients with FMS. The meta-analysis also brings to light that there is a clear need for further delineation of the duration and subtypes of BT, as the current data assessing this are limited. Larger-sample RCTs with similar treatment criteria, a longer follow-up time, and established evaluation criteria are needed.

## Figures and Tables

**Table 1:** Search strategy and results.

Database	Step	Search Algorithm	Items Found
PubMed	#1	“fibromyalgia” [Mesh]	8416
	#2	“musculoskeletal disease”	1718
	#3	“chronic pain syndrome”	675
	#4	“FMS”	9012
	#5	#1 OR #2 OR #3 OR #4	21,981
	#6	“balneotherapy”	1397
	#7	“spa therapy”	314
	#8	“thermal water”	298
	#9	“balneology”	6116
	#10	“BT”	23,370
	#11	#6) OR #7 OR #8 OR #9 OR #10	25,239
	#12	#5 AND #11	99
Embase	#1	‘fibromyalgia’/exp	20,842
	#2	‘musculoskeletal disease’	35,937
	#3	‘chronic pain syndrome’	1133
	#4	‘FMS’	12,213
	#5	#1 OR #2 OR #3 OR #4	67,541
	#6	“balneotherapy”/exp	16,620
	#7	“spa therapy”	602
	#8	“thermal water”	484
	#9	“balneology”	1471
	#10	“BT”	35,106
	#11	#6 OR #7 OR #8 OR #9 OR #10	52,736
	#12	#5 AND #11	434
Cochrane	#1	MeSH descriptor: [fibromyalgia] explode all trees	2927
	#2	musculoskeletal disease: ti,ab,kw (Word variations have been searched)	4114
	#3	chronic pain syndrome: ti,ab,kw (Word variations have been searched)	280
	#4	FMS: ti,ab,kw (Word variations have been searched)	649
	#5	#1 or #2 or #3 or #4	7576
	#6	MeSH descriptor: [balneotherapy] explode all trees	261
	#7	spa therapy: ti,ab,kw (Word variations have been searched)	122
	#8	thermal water: ti,ab,kw (Word variations have been searched)	79
	#9	nalneology: ti,ab,kw (Word variations have been searched)	211
	#10	BT: ti,ab,kw (Word variations have been searched)	1373
	#11	#6 or #7 or #8 or #9 or #10	1848
	#12	#5 and #11 restricted as clinical trials	52
Medline	#1	“fibromyalgia”	6926
	#2	“musculoskeletal disease”	6294
	#3	“chronic pain syndrome”	329
	#4	“FMS”	7109
	#5	fibromyalgia OR musculoskeletal disease OR chronic pain syndrome OR FMS	26,976
	#6	“balneotherapy”	472
	#7	“spa therapy”	273
	#8	“thermal water”	2131
	#9	“nalneology”	562
	#10	“BT”	21,713
	#11	nalneotherapy OR spa therapy OR thermal water OR balneology OR BT	38,850
	#12	#5 and #11	91
PsycINFO	#1	“fibromyalgia”	46
	#2	“musculoskeletal disease”	26
	#3	“chronic pain syndrome”	47
	#4	“FMS”	19
	#5	fibromyalgia OR musculoskeletal disease OR chronic pain syndrome OR FMS	116
	#6	“nalneotherapy”	0
	#7	“spa therapy”	1
	#8	“thermal water”	13
	#9	“nalneology”	1
	#10	“BT”	0
	#11	nalneotherapy OR spa therapy OR thermal water OR balneology OR BT	15
	#12	#5 and #11	1
Clinicaltrials.gov	#1	“fibromyalgia”	958
	#2	“musculoskeletal disease”	17,031
	#3	“chronic pain syndrome”	44
	#4	“FMS”	1026
	#5	fibromyalgia OR musculoskeletal disease OR Chronic pain syndrome OR FMS	17,308
	#6	“balneotherapy”	16
	#7	“spa therapy”	22
	#8	“thermal water”	3
	#9	“balneology”	6
	#10	“BT”	45
	#11	balneotherapy OR spa therapy OR thermal water OR balneology OR BT	107
	#12	balneotherapy OR spa therapy OR thermal water OR balneology OR BT|fibromyalgia OR musculoskeletal disease OR chronic pain syndrome OR FMS	207

**Table 2:** Detailed information and characteristics of included studies.

Studies	Country	Age (y)		Female/Male		Sample Size (*n*)		Duration		Follow-Up (w)
		BT	con	BT	con	BT	con	BT	con	
Fioravanti et al., 2018 [32]	Italy	56.16 ± 8.74	55.9 ± 6.61	48/2	47/3	50	50	16.02 ± 12.58 m	15.08 ± 10.87 m	24
Fernández et al., 2019 [33]	Spain	52.4 ± 8.6	53.4 ± 11.3	23/2	25/0	25	25	NA	NA	12
Dönmez et al., 2005 [26]	Turkey	43.3 ± 7.5	43.1 ± 6.9	NA	NA	16	13	11.5 ± 8.5 y	11.8 ± 7.5 y	36
Özkurt et al., 2012 [30]	Italy	50.8 ± 6	46.87 ± 8.8	21/0	24/0	21	24	12.9 ± 7 y	11.29 ± 6.2 y	12
Zijlstra et al., 2005 [34]	The Netherlands	48	47	55/3	73/3	58	76	10 m	10 m	48
Bağdatlı et al., 2015 [29]	Turkey	45.17 ± 9.09	42.77 ± 9.59	35/0	35/0	35	35	8.83 ± 4.74 y	8.37 ± 5.49 y	12
Evcik et al., 2002 [25]	Turkey	42 ± 6.8	41.5 ± 7.1	16/6	15/5	22	20	15.5 ± 7.2 m	14.1 ± 8.7 m	24
Ardıç et al., 2007 [27]	Turkey	43.5 ± 10.2	48.8 ± 8.8	NA	NA	12	9	23.4 ± 21.5 m	30.6 ± 28.6 m	3
Yurtkuran et al., 1996 [8]	Turkey	37.5	33.4	19/1	18/2	20	20	NA	NA	6
Fioravanti et al., 2007 [31]	Italy	46.2 ± 10.5	48.6 ± 9.4	39/1	39/1	40	40	2.21 ± 1.35 y	2.30 ± 1.42 y	16
Koçyiğit et al., 2016 [28]	Turkey	42.45 ± 9.93	41.77 ± 10.5	31/0	30/0	31	30	73.65 ± 59.15 m	69.40 ± 40.1 m	24

BT, balneotherapy; con, control; NA, not available; y, years; m, months; w, weeks.

**Table 3:** Detailed information and characteristics of balneotherapy.

Studies	Intervention		Duration (min)	Sessions (w)	Temperature (°C)	Ingredients
	BT	con				
Fioravanti et al., 2018 [32]	Vetriolo’s water	Tap water	15	2	36	Highly mineralized, pH 5.7, sulfate, calcium, magnesium, and iron
Fernández et al., 2019 [33]	Bicarbonate sodium water	Pharmacological	30	2	38	Medium mineralization, alkaline, lithic, fluorinated, silicated
Dönmez et al., 2005 [26]	Thermal water	Pharmacological	20	2	36 ± 1	Sodium, chlorine, bicarbonate and fluoride
Özkurt et al., 2012 [30]	Thermal baths	Pharmacological	20	2	36 ± 1	Sodium, chloride, and calcium with a total mineralization of 3367 mg/L
Zijlstra et al., 2005 [34]	Seawater	NA	NA	2^1/2^	NA	NA
Bağdatlı et al., 2015 [29]	Pool baths and mud pack	Exercise, education, and pharmacological	20	2	38	NA
Evcik et al., 2002 [25]	Thermal baths	Pharmacological and exercise	20	3	36	NA
Ardıç et al., 2007 [27]	Thermal pool water	Exercise or walking	20	3	36	NA
Yurtkuran et al., 1996 [8]	Therapeutic pool	Relaxation exercises	20	2	37	HCO_3_^−^, Cl^−^, F^−^, SO_4_^−2^, Ca^+2^, Mg^+2^, K^+^, Li^+1^
Fioravanti et al., 2007 [31]	Mud packs and thermal baths	Pharmacological	15	2	37–38	NA
Koçyiğit et al., 2016 [28]	Thermomineral water	Education	20	4	34.8	Na, K, Mg, Ca, F, CI, Bromur, I, NO_2_, NO_3_, SO_4_, HCO_3_, S, HPO_4_

BT, balneotherapy; con, control; w, week; NA, not available.

**Table 4:** GRADE summary of findings.

Outcome	Certainty Assessment							No. of Patients		Effect		Certainty
	No. of Studies	Study Design	Risk of Bias	Inconsistency	Indirectness	Imprecision	Other Considerations	Balneotherapy	Control	Relative(95% CI)	Absolute (95% CI)	
Pain at 2 weeks	5	Randomized trials	Serious	Very serious	Not serious	Not serious	None	138	137	-	SMD 0.92 lower (1.13 lower to 0.53 lower)	⨁◯◯◯ VERY LOW
Pain at 3 months	6	Randomized trials	Serious	Not serious	Not serious	Not serious	None	211	228	-	SMD 0.45 lower (0.73 lower to 0.16 lower)	⨁⨁⨁◯ MODERATE
Pain at 6 months	5	Randomized trials	Serious	Serious	Not serious	Not serious	None	177	189	-	SMD 0.7 lower (1.34 lower to 0.05 lower)	⨁⨁◯◯ LOW
FIQ at 2 weeks	7	Randomized trials	Serious	Serious	Not serious	Not serious	None	190	186	-	SMD 1.01 lower (1.43 lower to 0.59 lower)	⨁⨁◯◯ LOW
FIQ at 3 months	7	Randomized trials	Serious	Serious	Not serious	Not serious	None	236	253	-	SMD 0.64 lower (0.95 lower to 0.33 lower)	⨁⨁◯◯ LOW
FIQ at 6 months	4	Randomized trials	Serious	Very serious	Not serious	Not serious	None	155	169	-	SMD 0.59 lower (0.91 lower to 0.27 lower)	⨁◯◯◯ VERY LOW
TPC at 2 weeks	3	Randomized trials	Serious	Very serious	Not serious	Not serious	None	57	57	-	SMD 0.9 lower (2.02 lower to 0.23 higher)	⨁◯◯◯ VERY LOW
TPC at 3 months	4	Randomized trials	Serious	Not serious	Not serious	Not serious	None	126	143	-	SMD 0.47 lower (0.71 lower to 0.22 lower)	⨁⨁⨁◯ MODERATE
TPC at 6 months	4	Randomized trials	Serious	Very serious	Not serious	Not serious	None	127	139	-	SMD 0.89 lower (1.85 lower to 0.07 higher)	⨁◯◯◯ VERY LOW
BDI at 2 weeks	4	Randomized trials	Serious	Not serious	Not serious	Not serious	None	84	81	-	SMD 0.32 lower (0.63 lower to 0.01 lower)	⨁⨁⨁◯ MODERATE
BDI at 3 months	4	Randomized trials	Serious	Serious	Not serious	Not serious	None	133	148	-	SMD 0.23 lower (0.64 lower to 0.17 higher)	⨁⨁◯◯ LOW
BDI at 6 months	3	Randomized trials	Serious	Not serious	Not serious	Not serious	None	96	109	-	SMD 0.45 lower (0.73 lower to 0.17 lower)	⨁⨁⨁◯ MODERATE

CI: confidence interval; SMD: standardized mean difference; TPC, Tender Points Count; BDI, Beck’s Depression Index. ⨁⨁⨁⨁: High quality—We are very confident that the true effect lies close to that of the estimate of the effect; ⨁⨁⨁◯: Moderate quality—We are moderately confident in the effect estimate: the true effect is likely to be close to the estimate of the effect, but there is a possibility that it is substantially different; ⨁⨁◯◯: Low quality ()—Our confidence in the effect estimate is limited: the true effect may be substantially different from the estimate of the effect; ⨁◯◯◯: Very low quality—We have very little confidence in the effect estimate: the true effect is likely to be substantially different from the estimate of effect

## Data Availability

The data presented in this study are available in Figure 4, Figure 5, Figure 6, Figure 7, Figure 8 and Figure 9 and Table 2 and Table 3.
